# Rove beetles use fox scats as hunting grounds: Cryptic interactions, fatal attractions, and the structure of food webs

**DOI:** 10.1002/ecy.70391

**Published:** 2026-05-05

**Authors:** Mauriel Rodriguez Curras, Jonathan N. Pauli

**Affiliations:** ^1^ Department of Forest and Wildlife Ecology, Russell Labs University of Wisconsin‐Madison Madison Wisconsin USA; ^2^ Present address: Department of Environmental Science, Policy, and Management University of California Berkeley Berkeley California USA

Ecology is full of fleeting interactions that nevertheless can reveal the rules structuring communities and ecosystems. These cryptic interactions—subtle, ephemeral, and often context‐dependent—are frequently overlooked (Janzen, 1974) yet can illustrate underappreciated links between species and guilds. Indeed, cross‐guild, parallel interactions can reveal the fundamental mechanism driving trophic interactions that transcend spatial and temporal scales and taxa (Schmitz, 2005). Here, we document a cryptic and ephemeral food chain linking foxes, berries (an important food source for foxes in summer), scat, flies, and rove beetles—an interaction structure that differs markedly from classical producer–consumer–predator food chains.

For nearly a decade, our research team has conducted fieldwork on Isle Royale National Park in Lake Superior, United States, to investigate the trophic and behavioral effects of gray wolves (*Canis lupus*) on meso‐carnivores, namely, red foxes (*Vulpes vulpes*) and American martens (*Martes americana*; Rodriguez Curras et al., 2024). Across approximately 750 km of trails hiked each year, we collect fox and marten scats for genetic and dietary analysis (Lacin Alas et al., 2025) while subsequently trapping foxes to monitor their behavior, diet, and activity patterns. On September 10, 2021, we were fortunate to observe one of those “cryptic interactions that often escape the eye” (Janzen, 1974): prior to collecting a fox scat on the Feldtmann Trail near Cumberland Point (Figure 1A) we observed a gold‐and‐brown rove beetle (*Ontholestes cingulatus*)—also called “carrion beetles” (hereafter rove beetle)—perched on top of the scat with its brightly colored golden‐yellow tail waving in the air as a small mass of hovering flies circled above. As a large black fly of an unidentified species (Family: Sarcophagidae) descended, the rove beetle leapt up approximately 10 cm, captured, killed, and began consuming the fly (Figure 1B).

**FIGURE 1:**
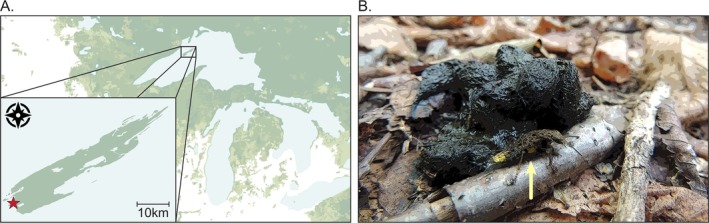
(A) Map of Isle Royale National Park, Michigan, United States, centered in the Great Lakes where our initial observation was made (red star). (B) A gold‐and‐brown rove beetle (*Ontholestes cingulatus*; gold arrow) waiting to ambush hovering flies of unidentified species next to a red fox (*Vulpes vulpes*) scat on Isle Royale National Park. Photograph taken by Mauriel Rodriguez Curras on September 10, 2021, near Cumberland Point, Isle Royale.

The following year, we set out to document rove beetles along the scat transects and en route to fox trapping locations. Out of a total of 148 red fox scats (all ≤4 days old, as transects were surveyed at 4‐day intervals) encountered and collected in the field along approximately 700 km of trail, we (along with two field technicians) detected rove beetles on approximately 10% of the total fox scats (*N* = 15). On two occasions, we detected two individuals on a single scat, one usually larger (estimated ~50% larger) than the other. In addition to the documented predation event reported here, we observed similar hunting behavior on two other occasions, although these were not photographed. We therefore distinguish between a single documented predation event and repeated observations of beetle presence on fox scats. However, we did not observe rove beetles on the scats of any other species (wolf, marten, or moose) that we encountered during our transects, including >30 moose droppings (though their larvae have been previously observed hatching from moose droppings in Isle Royale; Egan & Moon, 2018). Although fox scats commonly contain fruit material (>80%; Lacin Alas et al., 2025), we did not quantify fruit content in individual scats where beetles were observed. Thus, we present the proposed link between carnivore scats, fly attraction, and beetle predation as a hypothesis for future study. Following our observations, we reviewed all available publications in Web of Science, Scopus, and ScienceDirect using “*Ontholestes cingulatus*” as a keyword (*N* = 10), we found no documentations of this species utilizing terrestrial carnivore scats as a hunting ground, though they have been observed on river otter (*Lontra canadensis*) latrines in New Brunswick, Canada (Gallant et al., 2022). We expanded our search to include the family (*Staphylinidae*) AND “hunting” (yielding *N* = 169 records) and found records of other species (e.g., *Leistotrophus versicolor*) using two main tactics: ambushing flies at scat/carrion or on decomposing matter and prey‐luring by releasing scented abdominal secretions (Forsyth & Alcock, 1990; Lacin Alas et al., 2025).

Adult rove beetles are ambush predators that rely on the irregularly distributed resources of their prey, specifically, those that exploit ephemeral dung, carrion, or fungi (e.g., *Drosophila* spp.) as a hunting ground (Teskey, 1969; Worthen, 1989a). Our observation complements prior work on scat‐associated predator communities (e.g., Forsyth & Alcock, 1990; Hanski & Cambefort, 1991) and extends them by documenting predation on adult flies at carnivore scats. Rove beetles have been observed on fruiting mushrooms, livestock dung (e.g., cow *Bos taurus*), and ungulate carcasses where diverse fly communities compete for resources (food and ovipositing; Teskey, 1969; Worthen, 1989a, 1989b). In one experiment controlling the presence of rove beetles on a food source, face flies (*Musca autumnalis*) ceased oviposition or flew away if a rove beetle was detected (Teskey, 1969). Worthen (1989a), (1989b), showed that rove beetles structure fly communities via keystone predation, decreasing the natal recruitment of competitively dominant species and thereby releasing subordinate competitors from intraspecific competition. Within invertebrate food webs, rove beetles serve as voracious predators of ovipositing insects (e.g., *Drosophila* spp.), structuring communities and potentially providing feedback loops into vertebrate food webs, a hypothesis that warrants future testing.

Vertebrate scats are an important resource for a variety of plant and invertebrate species, serving as a mode of dispersal and biogeochemical cycling as well as a nutritional resource and microhabitat, and—as we described above—can facilitate trophic interactions. For example, due to their large size, the seeds of the jicaro (*Crescentia alata*) and guanacaste (*Enterolobium cyclocarpum*) in Central and South America were likely dispersed by large mammals of approximately 500 kg (Janzen & Martin, 1982). Alternatively, African dung beetles (Coleoptera: Scarabaeidae) predominantly rely on vertebrate dung, such as from African elephant (*Loxodonta africana*), and utilize these resources as ovipositing sites, providing refuge and resources for their offspring (Anderson & Coe, 1974). Our observation shows that beyond providing a direct function (e.g., dispersal or resources), vertebrate scats can facilitate important ecological interactions (i.e., predation), highlighting a crucial intersection between vertebrate and invertebrate guilds (Figure 2).

**FIGURE 2:**
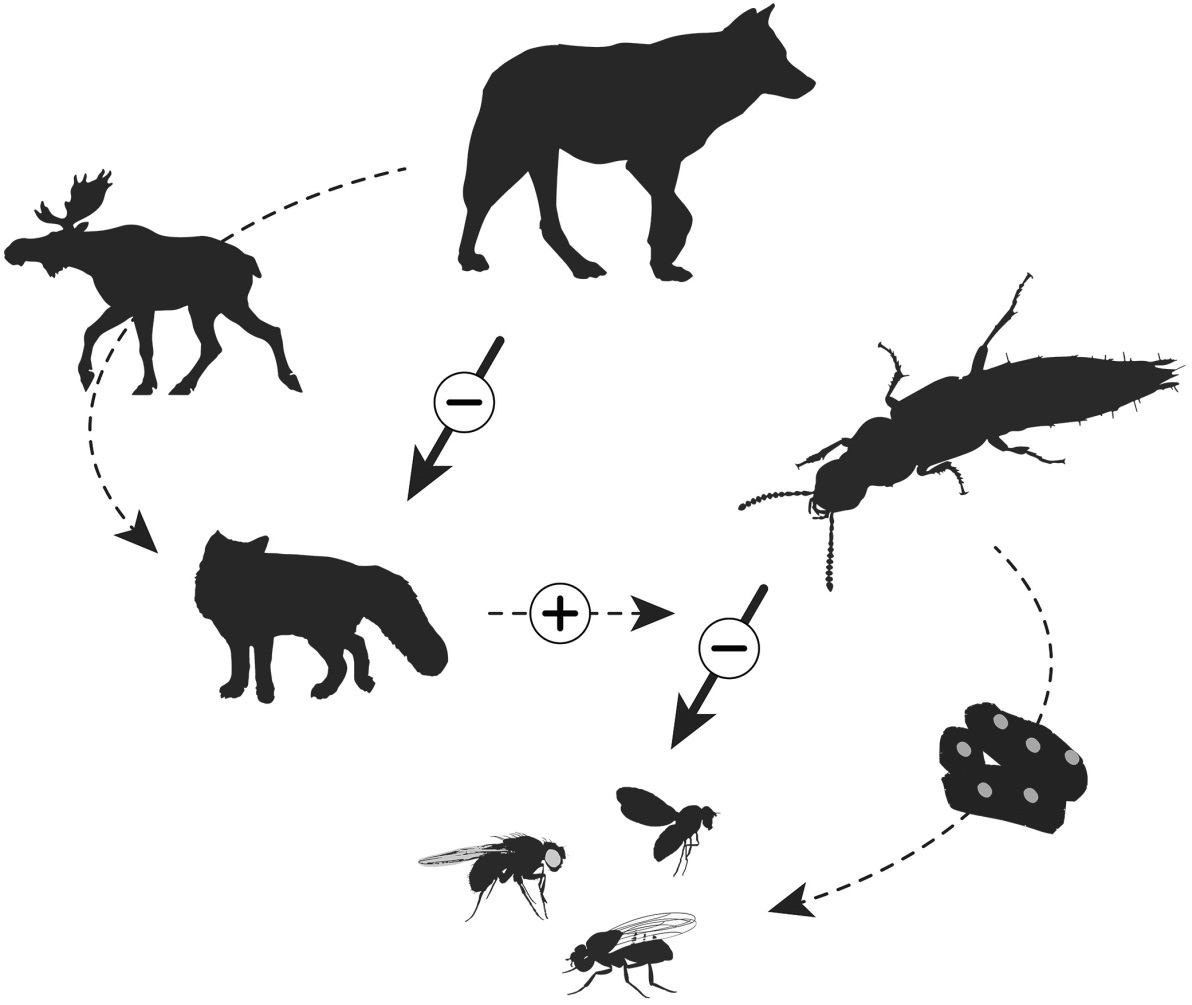
Parallel fatal attraction in vertebrate (wolf [*Canis lupus*], moose [*Alces alces*] carrion, and red fox [*Vulpes vulpes*]) and invertebrate (gold‐and‐brown rove beetle [*Ontholestes cingulatus*], fox scat, and ovipositing flies [Order: Diptera]) food webs. Moose carrion and fox scats serve as ephemeral resources of high nutritional demand, while wolves and rove beetles pose a predation risk to foxes and flies, respectively. Red foxes facilitate predation by rove beetles by creating an ephemeral resource (scat) for their primary prey (ovipositing flies; horizontal dashed line). Image credits: Scat was AI‐generated in Adobe Illustrator by the authors; *Vulpes* silhouette by painting Guo (CC BY 4.0; https://creativecommons.org/licenses/by/4.0/) was obtained from PhyloPic.org; the remaining silhouettes were obtained from PhyloPic.org under public domain licenses.

The fatal attraction hypothesis—originally formulated in the context of carnivore community ecology (Sivy et al., 2017)—posits that some resources may serve as a focus of attraction for species that can increase encounter rates with predators (or interspecific competitors) and thereby the likelihood of predation. Like other meso‐carnivores, foxes are attracted to wolf‐killed moose (*Alces alces*) carcasses to scavenge. The carcass creates a fatal attraction whereby foxes can experience a higher risk of encounter, and mortality, from wolves. Like foxes at carcasses, flies may experience a higher risk of predation from rove beetles at fox scats as they are eating or ovipositing. Foxes consume high proportions of berries (e.g., Sarsparilla *Aralia nudicaulis*, raspberry *Rubus idaeus*, and thimbleberry *Rubus parviflorus*), which have been historically found in >80% of all scats (Lacin Alas et al., 2025). As an ephemeral resource, the high consumption of berries in fox diets is likely to attract flies to a higher degree than ungulate or other carnivore scats. Rove beetles, then, can cue in on fox scats to use as a hunting ground for their potential prey as they swarm and oviposit. In this analogy, the fox scats represent the carrion in vertebrate food webs, while the interactions between the rove beetles−scat−flies liken to wolves−carrion−foxes (Figure 2). In both cases, ephemeral vertebrate resources (carcasses or scats) create focal points of attraction that elevate predation risk for consumers.

Predators play key roles in communities and ecosystems by regulating the populations of their prey, contributing to nutrient dispersal and cycling, and mediating the abundances and behaviors of subordinate competitors. Although there has been a recent emphasis on understanding the nested and modular interactions that mammalian predators facilitate, their impact on the interactions of invertebrates are seldom considered. A number of interesting, albeit cryptic, nested interactions between carnivores and invertebrates have recently been described, including Ethiopian wolves (*Canis simensis*) pollinating Ethiopian red hot poker (*Kniphofia foliosa*) flowers as they consume their nectar, likely competing with avian and invertebrate nectivores (Lai et al., 2024), and gray wolves consuming grasshoppers when their populations irrupt, putatively mediating competition between vertebrate and invertebrate herbivores (Barton et al., 2020). Our observation of foxes mediating predation by rove beetles in Isle Royale highlights a cryptic link between vertebrate and invertebrate food webs. While predator–prey and competitive interactions directly facilitate invertebrate populations by providing year‐round resources in the form of carrion, our observation exemplifies an alternative pathway by which vertebrate (e.g., carnivores) and invertebrate communities are linked via underlying mechanisms that structure food webs (Schmitz, 2005), highlighting an alternative, cross‐guild pathway through which vertebrate and invertebrate communities may be linked (Pringle & Hutchinson, 2020).

The ecological implications of parallel interactions across trophic guilds suggest that despite their taxonomic differences, vertebrate and invertebrate systems share many similarities in the form and function of ecological interactions (Meadows et al., 2017). For example, the direct and indirect effects of predation have previously been described as a key parallel between vertebrates and invertebrates, serving as fundamental mechanisms structuring communities and ecosystems (Schmitz, 2005). Indeed, rove beetles not only pose direct control on flies through predation, but they can exert an indirect cost through impacts on recruitment (Worthen, 1989b). The direct and indirect regulation that rove beetles pose on fly communities is consistent with small‐scale experiments featuring spiders (Arachnids; Schmitz, 2008) and large‐scale studies featuring pumas (*Puma concolor*; Laundre et al., 2010), which have suggested that ambushing predators can have strong effects on prey densities and behaviors, thereby influencing community structure and ecosystem function (Preisser et al., 2007).

Cryptic interactions, specifically those that link modular food webs, may influence interspecific interactions across guilds (McCann et al., 1998). Invertebrate communities, and specifically flies, have important direct and indirect impacts on vertebrate populations through competition for resources, as pests, pollination services, and disease/parasite transmission. Predators such as rove beetles, then, may regulate invertebrate prey in ways that could cascade to vertebrates—an ecological consequence we propose as a testable hypothesis rather than a demonstrated outcome of the present study.

Our observation of rove beetles preying on flies at red fox scats reveals a cryptic connection between vertebrate and invertebrate food webs. Such cross‐guild interactions highlight how ephemeral vertebrate resources can structure invertebrate communities and, further, exemplify the cascading and facultative nature of species interactions. Just as carrion shapes vertebrate predator–scavenger dynamics, fox scats act as microhabitats that attract, concentrate, and expose invertebrate prey to predation. This parallel—fatal attractions (sensu; Sivy et al., 2017)—underscores that the form and function of trophic interactions are remarkably conserved across taxonomic boundaries. Our findings highlight that vertebrate and invertebrate food webs are not silos but interwoven, dynamic components of food webs, and that attention to these subtle mechanisms can yield new insights into the structural connections that link otherwise disparate components of ecosystems.

## CONFLICT OF INTEREST STATEMENT

The authors declare no conflicts of interest.
